# A Case Report of Metastatic Melanoma of Unknown Primary with Massive Jejunal Involvement Mimicking Intestinal Lymphoma in a Young Adult: Diagnostic Pitfalls and Surgical Challenges

**DOI:** 10.3390/reports9030219

**Published:** 2026-07-10

**Authors:** Alexandra Caziuc, Radu Alexandru Ilieș, George Ionuț Golea, Cristian-Florin Bibu-Monuș, Andrada Larisa Deac, George Călin Dindelegan

**Affiliations:** 1 Department of General Surgery, “Iuliu Hațieganu” University of Medicine and Pharmacy, 400012 Cluj-Napoca, Romania; caziuc.alexandra@umfcluj.ro (A.C.); george.dindelegan@umfcluj.ro (G.C.D.); 2 First Surgical Unit, Emergency County Hospital Cluj, 400006 Cluj-Napoca, Romania; cristian.flor.bibumonus@elearn.umfcluj.ro; 3 Faculty of Medicine, “Iuliu Hațieganu” University of Medicine and Pharmacy, 400012 Cluj-Napoca, Romania; 4 Department of Pharmacology, Toxicology and Clinical Pharmacology, “Iuliu Hațieganu” University of Medicine and Pharmacy, 400012 Cluj-Napoca, Romania; deac.andrada@umfcluj.ro

**Keywords:** malignant melanoma, small intestine, bowel obstruction, immunotherapy

## Abstract

**Background and Clinical Significance**: Malignant melanoma with primary or metastatic intestinal involvement is a rare entity, often diagnosed late and associated with severe complications such as bowel obstruction and perforation. Differential diagnosis of primary intestinal lymphoma may be challenging in the absence of an identifiable primary lesion. **Case Presentation**: We report the case of a 35-year-old male with no significant medical history who was admitted for persistent abdominal symptoms. Contrast-enhanced abdominal CT revealed a giant circumferential jejunal mass (109/147/156 mm) causing marked luminal stenosis and mesenteric lymphadenopathy, initially raising suspicion of primary intestinal lymphoma. The patient subsequently developed upper intestinal obstruction and severe anemia (Hb 5.5 g/dL), requiring an emergency exploratory laparotomy. Intraoperatively, a voluminous unresectable tumor extending to the mesenteric root was identified, and a feeding jejunostomy was performed. The postoperative course was complicated by tumor perforation and generalized peritonitis, necessitating reoperation. Histopathological examination established the diagnosis of malignant melanoma, with no identifiable primary site, which is most consistent with metastatic melanoma (MUP). PET-CT staging demonstrated metastatic disease (mesenteric, retroperitoneal and supraclavicular lymph nodes, as well as subcutaneous nodules), consistent with a stage IV disease. Molecular analysis revealed a BRAF V600E mutation. Combined immunotherapy (Nivolumab + Ipilimumab) was initiated, resulting in a partial radiological response after three cycles. **Conclusions**: Intestinal involvement by malignant melanoma might mimic other gastrointestinal malignancies and be the cause of a delayed diagnosis and severe surgical complications. Multidisciplinary management is essential, and modern immunotherapy offers promising outcomes even in advanced-stage disease.

## 1. Introduction and Clinical Significance

Malignant melanoma represents approximately 1–3% of all gastrointestinal malignancies, with the majority of cases corresponding to metastatic rather than primary tumors. Both primary and metastatic intestinal melanomas are characterized by increased biological aggressiveness and a less favorable prognosis in comparison to melanomas arising in other anatomical locations. Early-stage, asymptomatic metastatic involvement of the gastrointestinal tract is infrequently reported, as only 1–5% of cases are detected incidentally during imaging investigations. Such incidental diagnoses are generally associated with improved therapeutic prospects [1].

Gastrointestinal metastases originating from melanoma with an unknown primary cutaneous lesion account for approximately 4–9% of cases. In spite of advances in diagnostic techniques, only 1.5–4.4% of gastrointestinal metastatic lesions are diagnosed during the patient’s lifetime [2].

Melanoma may develop at any level of the gastrointestinal mucosa. However, it most frequently involves the anorectal region, particularly the rectum (22.2%) and anal canal (31.4%), followed by the oropharynx (32.8%). Less commonly affected sites include the esophagus (5.9%), stomach (2.7%), small intestine (2.3%), gallbladder (1.4%), and colon (0.9%) [3].

Primary intestinal melanoma is an exceptionally rare entity, diagnosed in the absence of a prior history of cutaneous melanoma [4,5,6,7]. It usually affects middle-aged to elderly patients and most commonly involves the duodenum, jejunum, or ileum [4,8]. Clinically, patients may present with anemia, obscure gastrointestinal bleeding, abdominal pain, obstruction, or intussusception [6,7]. Lesions are usually solitary and may appear endoscopically as ulcerated or polypoid masses that are frequently amelanotic in nature [4,9]. Imaging studies often reveal focal bowel wall thickening or a discrete mass, sometimes associated with intussusception [5,7]. Histopathological examination confirms melanoma that is often amelanotic, with immunohistochemical positivity for S100, HMB-45, and Melan-A [4,10]. By definition, extraintestinal disease is absent at diagnosis and the diagnosis is established by exclusion according to the Sachs criteria [4,5,11]. Surgical resection, with or without adjuvant therapy, represents the main therapeutic approach, and prognosis may be more favorable when complete resection is achieved [4,5].

In contrast, metastatic intestinal melanoma is more common compared to the primary form and is usually associated with a known, sometimes remote, history of cutaneous melanoma [12,13,14,15]. It also predominantly affects middle-aged to elderly individuals, with a predilection for the jejunum and ileum [12,16,17]. Clinical manifestations include anemia, melena, bowel obstruction, intussusception, and late-stage gastrointestinal bleeding [15,16]. Unlike primary tumors, metastatic lesions are frequently multiple and may appear endoscopically as pigmented or ulcerated nodules or masses, often in a multifocal distribution [12,17]. Cross-sectional imaging, including CT or PET, commonly demonstrates multiple hypermetabolic lesions [12,17]. Histologically, these tumors are often pigmented melanomas, expressing S100, HMB-45, Melan-A, and SOX-10 on immunohistochemistry [14,16]. Extraintestinal disease is usually present at diagnosis or identified subsequently [13,17]. The diagnosis is supported by the detection of a known or newly identified primary melanoma [12,13]. Management typically involves surgical intervention for complications and is combined with systemic oncologic therapy, and the prognosis is generally poor because of disseminated disease [15,17].

This work aims to discuss a diagnostically challenging case of malignant melanoma with massive small bowel involvement, initially mimicking primary intestinal lymphoma, with an indeterminate primary origin. The current paper also has the objective of emphasizing a wide variety of aspects (clinical, radiological, surgical, histopathological, and molecular) that guided diagnosis and management.

## 2. Case Presentation

A 35-year-old male patient with no previous significant medical history was admitted in April 2025 to Cluj County Emergency Hospital, Cluj-Napoca, Romania, specifically the Second Department of Internal Medicine, due to persistent abdominal complaints. The patient had associated hepatic steatosis. He had no chronic medication use and no known drug allergies. A relevant recent history included an unintentional weight loss of approximately 15 kg over one month.

Contrast-enhanced abdominal computed tomography (CT) was performed on 3 April 2025, revealing a giant, circumferential mass (109/147/156 mm) located at the level of the jejunum, which caused marked luminal stenosis and significant compression on the surrounding structures (Figure 1). The lesion infiltrated the root of the mesentery and was associated with numerous mesenteric adenopathies. Based on these findings, initial suspicion was of primary intestinal lymphoma. No ascites or distant metastases were noted. The patient was discharged with their histopathological exam pending.

On 8 May 2025, the patient presented to the Emergency Department in the same hospital with worsening gastrointestinal symptoms, including nausea, persistent vomiting, diffuse abdominal pain, progressive abdominal distension, fatigue and dyspnea. Laboratory investigations revealed severe anemia (Hb 5.5 g/dL) with thrombocytosis, while hepatic and renal functions were preserved. Abdominal CT was performed and emphasized the intestinal mass, which was suspected to be of lymphomatous origin, associated with loco-regional lymphadenopathy (Figure 2). Multiple jejunal loops with a conglomerate appearance were identified adjacent to the mass, with the proximal loop dilated up to 50 mm and containing fluid. The lesion exerted a mass effect on both the ascending colon and the inferior vena cava. Abdominal ultrasound confirmed distended intestinal loops measuring up to 5 cm without peristalsis but with fluid stasis, findings which support the diagnosis of upper intestinal obstruction. The differences in tumor dimensions between the two CT examinations likely reflect disease progression and differences in imaging planes and measurement technique.

Because of the clinical and radiologic suspicion of intestinal obstruction in the context of a giant jejunal tumor, the patient underwent an emergency exploratory laparotomy in the First Department of Surgery. Intraoperatively, a voluminous tumor incorporating the third and fourth jejunal loops and extending towards the mesenteric root was observed (Figure 3 and Figure 4). Complete surgical resection was not feasible because of extensive mesenteric root involvement and locally advanced disease. Due to its unresectability and an initial suspicion of lymphoma, a feeding jejunostomy was performed (allowing subsequent oncologic treatment).

The initial postoperative course was favorable, with early extubation and hemodynamic stabilization. However, the patient developed persistent postoperative ileus requiring parenteral nutrition.

On postoperative day 4, the patient complained of severe abdominal pain accompanied by hypotension (signs of generalized peritonitis). Re-laparotomy was performed due to clinical deterioration and signs of peritonitis and it revealed perforation of the tumor with peritoneal contamination. An enterotomy was performed, followed by lateral jejunojejunostomy, after which the previously created jejunostomy was removed. A directed laparostomy was established, with multiple drains placed to ensure adequate evacuation of the collection. Overall, the postoperative evolution was favorable, with progressive formation of granulation tissue and gradual closure of the laparostomy (Figure 5).

Postoperatively, the patient developed septic shock requiring mechanical ventilation, vasopressor support with norepinephrine up to 0.25 µg/kg/min, and continuous renal replacement therapy. The clinical course was complicated by unfavorable events such as acute kidney injury, metabolic acidosis and oliguria. Blood and peritoneal fluid cultures confirmed Escherichia coli infection. Targeted antibiotic therapy was initiated and subsequently adjusted according to antibiogram results until the complete eradication of the infection. After a period of intensive care management, including renal replacement therapy (continuous veno-venous hemodiafiltration) and hemodynamic stabilization, the patient showed progressive clinical improvement.

Following clinical improvement, vasopressor support was discontinued, renal function recovered with restoration of diuresis, and the patient was successfully extubated. He demonstrated gradual clinical recovery with stabilization of respiratory and abdominal status.

Histopathological examination of the surgical specimen revealed multiple intestinal resection specimens, including a 2 cm whitish tumor-forming lesion within one of the small bowel segments.

Microscopically, the tumor presented diffuse proliferation of discohesive malignant cells with focal nesting and a trabecular architecture, infiltrating the full thickness of the small intestinal wall. The neoplastic cells exhibited marked nuclear pleomorphism, irregular hyperchromatic to vesicular nuclei, prominent eosinophilic nucleoli, and variable eosinophilic to pale cytoplasm, with occasional plasmacytoid features. Numerous mitotic figures were found. Extensive areas of ischemic tumor necrosis were present at the luminal surface. Lymphatic emboli were also observed, but no definite vascular or perineural invasion was identified. The serosal surface presented focal fibrino-neutrophilic exudate without evidence of tumor cell involvement.

Extensive examination of multiple sections of the intestinal mucosa did not identify any in situ or junctional melanocytic component at the mucosal interface.

Due to the fragmented and unoriented nature of the specimens, the resection margins could not be reliably assessed.

Immunohistochemical analysis showed diffuse strong positivity for S100, SOX10, Melan-A and PRAME, while pan-cytokeratin, EMA, and LCA were negative in tumor cells (with appropriate internal positive controls). This immunoprofile supported the diagnosis of malignant melanoma.

The histomorphological and immunohistochemical features were consistent with the diagnosis of malignant melanoma. However, based on histopathological examination alone, it was not possible to definitively distinguish between a primary small bowel melanoma and metastatic disease; therefore, clinical and radiological correlation was recommended.

Subsequent Dermatology, Ophthalmology and Ear, Nose, and Throat (ENT) evaluations were performed, all yielding normal findings with no detectable lesions.

Staging included PET-CT (6 June 2025), which indicated a large hypermetabolic abdominal mass (maximum SUV: 16.9), together with mesenteric and retroperitoneal lymphadenopathies (SUV up to 12.9), right supraclavicular nodal metastasis (28 × 18 mm, SUV: 13.5) and subcutaneous nodules. Brain MRI was negative for any lesions. The final diagnosis was stage IV malignant melanoma with small bowel involvement and disseminated disease.

Molecular analysis identified a BRAF V600E mutation. Consequently, the case was discussed in the multidisciplinary oncologic tumor board, which reviewed the imaging, histopathological, and molecular findings. Considering the diagnosis of stage IV malignant melanoma with a positive BRAF V600E mutation and the patient’s overall condition, the board recommended initiation of systemic immunotherapy. It was initiated in June 2025: Nivolumab (1 mg/kg) combined with Ipilimumab (3 mg/kg), followed by planned Nivolumab maintenance. No significant immune-related adverse events were observed during the reported treatment cycles. By the third cycle (28 July 2025), partial regression of the right supraclavicular lymph node (from 4.5 cm to 2 cm) was documented, while the general condition of the patient was stable.

A comprehensive summary of the histopathological, immunohistochemical, molecular, and imaging findings is presented in Table 1.

The absence of an in situ or junctional melanocytic component within the intestinal mucosa, together with the presence of disseminated disease on PET-CT, strongly favors a diagnosis of metastatic melanoma rather than a primary small bowel melanoma.

The chronological sequence of the clinical presentation, diagnostic work-up, surgical interventions, and oncologic treatment is summarized in Figure 6.

## 3. Discussion

The presented case illustrates both the diagnostic and therapeutic challenges that occurred in a young adult patient with intestinal melanoma. Initial imaging and intraoperative findings suggested another pathology (primary intestinal lymphoma), given the circumferential jejunal mass with the presence of luminal stenosis and extensive, bulky, mesenteric adenopathies. However, histopathology and immunohistochemistry established the diagnosis of malignant melanoma with small bowel involvement. Findings were most consistent with metastatic melanoma of unknown primary (MUP), rather than a primary intestinal melanoma, with important implications regarding the differential diagnosis between rare intestinal malignancies. Although melanoma typically affects older individuals, metastatic melanoma of unknown primary (MUP) can rarely present in younger patients [18]. In this age group, atypical gastrointestinal presentations may further complicate diagnostic pathways and contribute to an initial misdiagnosis, particularly when mimicking lymphoma or other primary gastrointestinal malignancies [18].

Histopathological examination confirmed the diagnosis of malignant melanoma which involved the small bowel. Dedifferentiated melanomas may lose expression of traditional markers such as Melan-A and even PRAME, while SOX10 often remains retained, highlighting the importance of using a broad immunohistochemical panel in diagnostically challenging cases [19,20].

Considering the absence of a previously known history of cutaneous, ocular, or mucosal melanoma, together with the negative findings on several evaluations (complete dermatologic, ophthalmologic, and ENT), a primary cutaneous or mucosal origin could not be identified. Still, subsequent staging with PET-CT showed disseminated disease, including mesenteric, retroperitoneal, and supraclavicular lymph node involvement, as well as subcutaneous metastases, consistent with stage IV systemic spread. In this context, the pattern of disease was most compatible with MUP, rather than a primary small bowel melanoma. Gastrointestinal involvement by melanoma in the setting of disseminated disease is more frequently metastatic [1,2], while primary intestinal melanoma remains an exceptional and strictly exclusion-based diagnosis that was improbable in the presented case (based on the absence of a primary lesion and the presence of disseminated disease on PET-CT, which fulfilled the criteria for MUP).

The main diagnostic pitfalls in the presented case include: (a) radiological and intraoperative mimicry of primary intestinal lymphoma, (b) amelanotic or non-specific presentation of gastrointestinal melanoma, (c) metastatic MUP and (d) potential diagnostic uncertainty due to tumor heterogeneity in advanced melanoma.

Historically, the diagnosis of primary gastrointestinal melanoma relied on the criteria proposed by Sachs et al. [21], which include: (a) the presence of a solitary intestinal lesion that is biopsy-proven; (b) no concurrent or prior cutaneous, mucosal or ocular melanoma; and (c) no evidence of extraintestinal disease at the time of diagnosis (after thorough clinical evaluation) [21,22]. In the present case, even if no primary lesion was identified on comprehensive dermatologic, ophthalmologic, and ENT assessments, the presence of disseminated disease on PET-CT imaging was not compatible with a primary gastrointestinal melanoma according to the aforementioned criteria.

Moreover, the surgical course of the case was particularly challenging. The patient required two separate emergency laparotomies within four days (initially due to intestinal obstruction and subsequently for perforation of the tumoral mass with generalized peritonitis). The extent of local invasion and the impossibility to perform complete resection are in accordance with the aggressive biological behavior of this disease (and the limitations of surgery in advanced intestinal melanoma).

On the whole, this case highlights the critical role of histopathology, immunohistochemistry, and molecular testing in establishing the final diagnosis, as well as the importance of multidisciplinary tumor board decision-making in complex oncologic surgical cases.

## 4. Conclusions

Intestinal melanoma represents a rare and aggressive malignancy, most frequently representing metastatic disease rather than a primary tumor. Differentiating metastatic intestinal involvement from other gastrointestinal malignancies can be challenging and requires a multidisciplinary approach combining imaging, histopathology, and molecular analysis.

In metastatic MUP, intestinal involvement is often associated with disseminated disease and a poor prognosis. Early recognition is essential to prevent life-threatening complications and to allow timely initiation of systemic therapy, including modern immunotherapy, which may achieve meaningful clinical responses even in advanced-stage diseases.

## Figures and Tables

**Figure 1:**
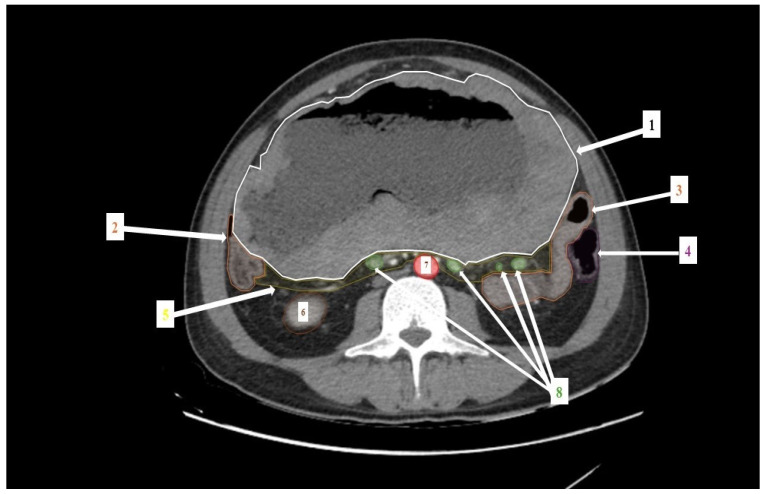
Contrast-enhanced abdominopelvic CT (axial view) demonstrating a giant circumferential jejunal mass (1), measuring 109 × 147 × 156 mm, causing marked luminal stenosis (2) and compression of adjacent structures, including the small bowel (3), descending colon (4), small bowel mesentery (5), right kidney (6), abdominal aorta (7), and multiple mesenteric lymph nodes (8). The imaging findings are highly suggestive of primary intestinal lymphoma.

**Figure 2:**
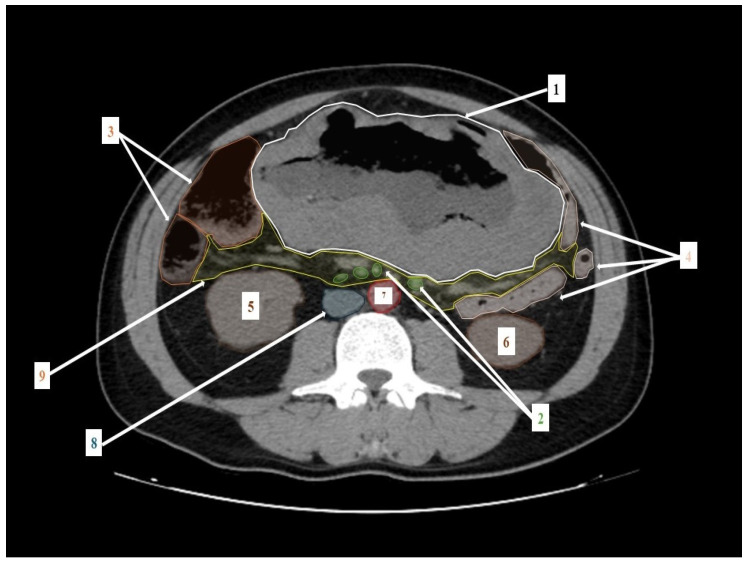
Contrast-enhanced axial CT demonstrating a large intestinal tumor (1), measuring 131 × 260 × 246 mm, with locoregional lymphadenopathy (2), proximal jejunal dilatation up to 50 mm with fluid stasis (3), agglutinated bowel loops (4), and mass effect on adjacent structures, including the right kidney (5), left kidney (6), abdominal aorta (7), inferior vena cava (8), and mesentery (9), resulting in intestinal obstruction.

**Figure 3:**
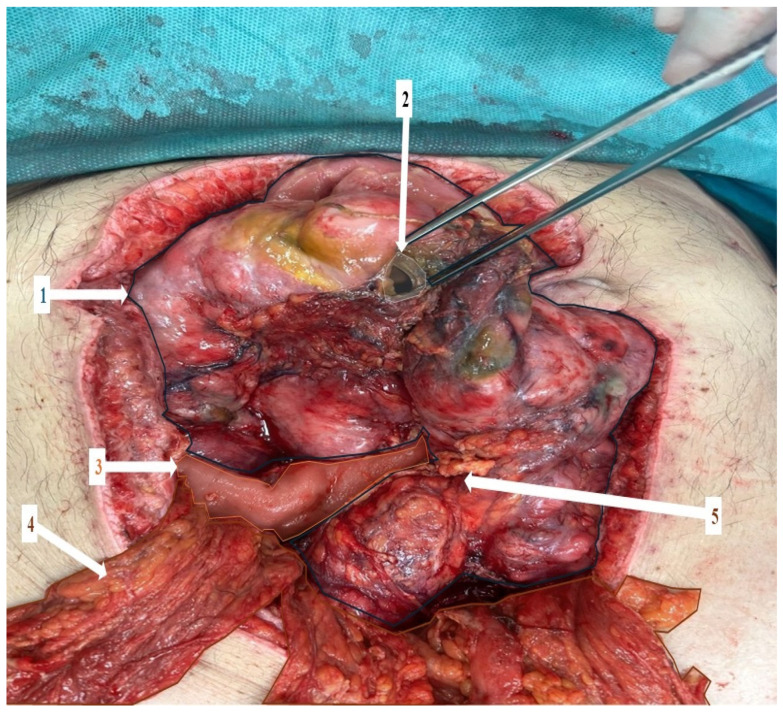
Intraoperative findings revealed a large jejunal tumor (1) with a perforation (2) on the anterior wall. The tumor extends into the mesenteric root (5). The distal jejunum (3) is intact and in continuity with the large tumor. The tumor does not invade the omentum (4).

**Figure 4:**
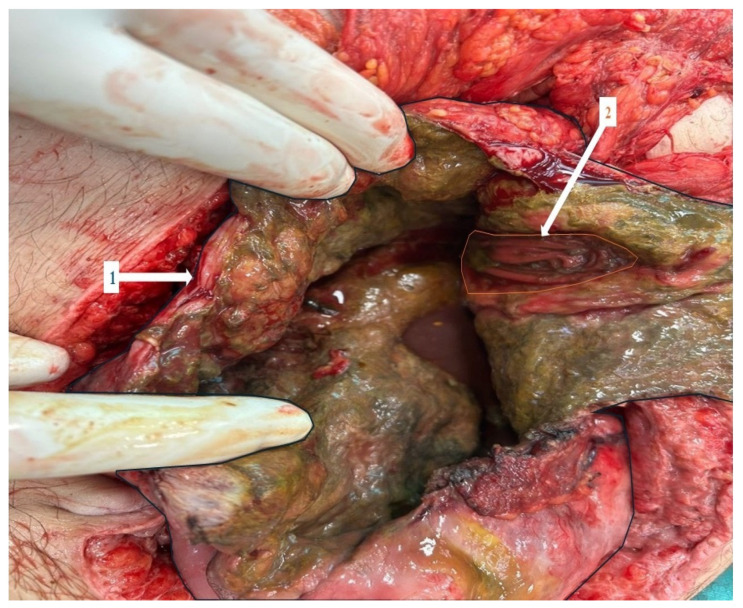
Intraoperative findings revealing the jejunal lumen (1) opening in the tumor cavity (2).

**Figure 5:**
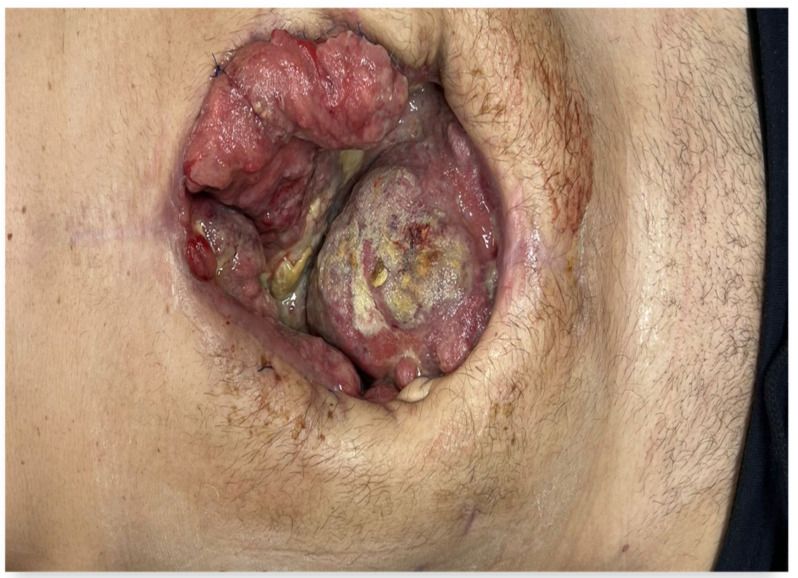
Postoperative evolution of the abdominal wound showing gradual closure of the laparostomy with progressive granulation tissue formation.

**Figure 6:**
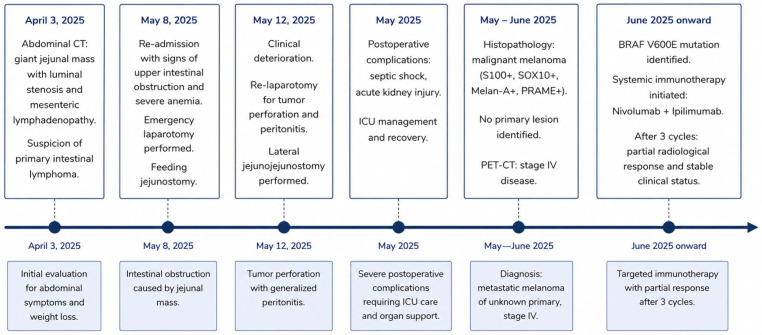
Timeline of the clinical presentation, surgical interventions, diagnostic work-up, and treatment course.

**Table 1:** Summary of histopathological, immunohistochemical, molecular, and imaging findings.

Category	Investigation	Findings
Histopathology	Small bowel surgical specimen	Diffuse proliferation of discohesive malignant cells with focal nesting and a trabecular architecture; full-thickness infiltration of the bowel wall; marked nuclear pleomorphism; prominent nucleoli; high mitotic activity; extensive tumor necrosis; lymphatic emboli present
Margins	Surgical specimen	Not assessable due to fragmented and unoriented tissue
Immunohistochemistry	S100	Diffuse strong positivity
	SOX10	Diffuse strong positivity
	Melan-A	Diffuse strong positivity
	PRAME	Diffuse strong positivity
	Pan-cytokeratin	Negative
	EMA	Negative
	LCA	Negative
Molecular testing	BRAF mutation	BRAF V600E-positive
Imaging: CT (3 April 2025)	Initial CT of the abdomen	Giant circumferential jejunal mass (109 × 147 × 156 mm), marked luminal stenosis, mesenteric lymphadenopathy, and no distant metastases
Imaging: CT (8 May 2025)	Follow-up CT of the abdomen	Enlarged jejunal mass (131 × 260 × 246 mm), proximal bowel dilatation (up to 50 mm), fluid stasis, agglutinated loops, and mass effect on adjacent organs
Imaging: PET-CT (6 June 2025)	Whole-body staging	Hypermetabolic abdominal mass (SUV max: 16.9), mesenteric/retroperitoneal and right supraclavicular lymph nodes (SUV up to 13.5), and subcutaneous metastases
Imaging: Brain MRI	CNS staging	No intracranial metastases

CT—computed tomography; PET-CT—positron emission tomography–computed tomography; MRI—magnetic resonance imaging; IHC—immunohistochemistry; S100—S100 calcium-binding protein; SOX10—SRY-box transcription factor 10; Melan-A—melanoma antigen recognized by T cells 1 (MART-1); PRAME—preferentially expressed antigen in melanoma; EMA—epithelial membrane antigen; LCA—leukocyte common antigen (CD45); BRAF V600E—activating mutation in the BRAF gene (V600E substitution); SUV—standardized uptake value; MUP—metastatic melanoma of unknown primary; CNS—central nervous system.

## Data Availability

The data presented in this study are available on request from the corresponding author due to privacy reasons.
